# Does increased serum d-lactate mean subclinical hyperpermeability of intestinal barrier in middle-aged nonobese males with OSA?

**DOI:** 10.1097/MD.0000000000009144

**Published:** 2017-12-08

**Authors:** Mulalibike Heizati, Nanfang Li, Liang Shao, Xiaoguang Yao, Yingchun Wang, Jing Hong, Ling Zhou, Delian Zhang, Guijuan Chang, Suofeiya Abulikemu

**Affiliations:** The Center of Hypertension of the People's Hospital of Xinjiang Uygur Autonomous Region China; The Center of Diagnosis, Treatment and Research of Hypertension in Xinjiang China.

**Keywords:** d-lactate., intestinal permeability, low-grade systemic inflammation, obstructive sleep apnea

## Abstract

Few attention has been directed to the potential effects of intermittent hypoxia experienced in obstructive sleep apnea on the integrity and permeability of intestinal barrier, particularly in adults. Therefore, we evaluated alteration in serum d-lactate concentration in middle-aged males with obstructive sleep apnea to value permeability of intestinal barrier. In this current cross-sectional study, consecutive 159 males were studied. Obstructive sleep apnea was determined by polysomnography and apnea hypopnea index ≥15 event/h was defined as obstructive sleep apnea. D-lactate, lipopolysaccharide binding protein, interleukin-1β, interleukin-6 and tumor necrosis factor-α by ELISA method. Nonobese obstructive sleep apnea (OSA) males showed significantly higher serum d-LA than did nonobese [1374.35 (816-1735) μg/L vs 1166.43 (730–1815) μg/L, *P = *.018], and obese non-OSA ones [1374.35 (816-1735) μg/L vs 1188.75 (736–1557) μg/L, *P = *.045], whereas serum LBP levels showed no differences within groups. Serum IL-1β was also slightly higher in nonobese OSA males, but with statistical significance, than in nonobese (19.39 ± 4.67 ng/L vs 17.25 ± 3.66 ng/L, *P = *.041), and obese non-OSA ones (19.39 ± 4.67 ng/L vs 17.42 ± 3.79 ng/L, *P = *.047), whereas other biomarkers, IL-6 and TNF-a did not show significant differences among groups. In stepwise multiple linear regression analysis, serum d-LA was independently positively associated with AHI (*B* = 5.577, *P = *.022), and ODI3 (*B* = 4.550, *P = *.024) and negatively with LSaO2 (*B* = −12.234, *P = *.019). Finally, we arrived at a conclusion that serum d-lactate was increased in nonobese middle-aged males with obstrutive sleep apnea, possibly suggesting existence of subclinical disruption of intestinal barrier, and showed significant associations with inflammatory mediators, possibly being involved in systemic inflammation of obstructive sleep apnea.

## 1 Introduction

Obstructive sleep apnea (OSA) is characterized by an intermittent repeatable cessation of airflow to the lung due to closure of the airway at pharyngeal level and afflicting about 24 to 42% adults^[1]^ and has been implicated as an independent risk factor for hypertension and cardio-cerebrovascular diseases,^[2]^ particularly among middle-aged males.^[3]^ Intermittent hypoxia, one of the physiological insults and the hallmarks of OSA, appears to play significant roles since it triggers inflammation and oxidative stress cascades that are deleterious and contribute to the multiorgan morbid consequences of OSA.^[4]^

A few attention has been directed to the potential effects of OSA on the integrity and permeability of intestinal barrier, particularly in adults,^[5]^ although its pathological impacts on different organs and tissues have been studied.^[6–8]^ In fact, several studies have implicated the disruption of intestinal barrier in the condition of hypoxia. First of all, high-altitude hypoxia causes severe damage to different organs, especially to the intestinal tract. The incidence of digestive system disease is quite high among high-altitude residents and immigrants.^[9]^ In addition, various lines of evidence have also implicated intestinal ischemia reperfusion injury, a significant problem experienced in main surgeries such as cardiopulmonary bypass, is associated with increased intestinal permeability and bacterial translocation into the portal and systemic circulation.^[10]^ Furthermore, recent data have linked systemic low-grade inflammation, another hallmark of OSA, in some chronic inflammatory conditions to the disturbances in the composition of the gut microbial flora and alterations in gut barrier, encompassing obesity, diabetes and nonalcoholic fatty liver disease (NAFLD).^[5,11–14]^

Therefore, we hypothesized that intestinal barrier might be subclinically disrupted during OSA, facilitating translocation of bacterial products into circulation. To this end, we assessed whether serum levels of d-lactate (d-LA)^[15–17]^ and lipopolysaccharide binding protein (LBP)^[5]^ were altered as biomarkers for disruption of intestinal barrier and systemic inflammatory biomarkers including interleukin-1β (IL-1β), interleukin-6 (IL-6), tumor necrosis factor-a (TNF-α), and high-sensitivity C-reactive protein (Hs-CRP).

## 2 Methods

### 2.1 Recruitment of subjects

This is a cross-sectional study. Consecutive 159 Han Chinese males, aged 30 to 65 years, clinically suspicious with OSA and referred to sleep monitoring for the first time, were recruited to the present study at the People's Hospital of Xinjiang Uygur Autonomous Region from April to December 2013. Ethics’ Committee of aforementioned hospital approved the study protocol. Written informed consents and questionnaires were obtained from all subjects before participation. Exclusion criteria of the present study encompassed: history of atherosclerotic disease (myocardial infarction or stroke < 6 months), congestive heart failure, diabetes, thyroid diseases, acute and chronic respiratory diseases, systemic infections (including acute and chronic intestinal diseases based on collection of clinical history and questionary), and a therapy for 4 weeks prior to study entry with inhaled, oral, or nasal steroids, or usage of other anti-inflammatory drugs. Those diagnosed as central sleep apnea were also excluded. Subjects who had contact with productive dust, poisonous gas and alcohol, and substance abuse were also excluded by personal history. Prospective process to recruit subjects with standard questionary by trained staff allowed us to exclude subjects with any signs of above conditions and thus made the exclusion criteria quite strict. A complete physical examination was performed, including neurologic, cardiopulmonary, abdominal and ENT examinations. Subjects who had stable co-morbidities were managed with appropriate medical therapy.

### 2.2 OSA evaluation

All participants underwent overnight attended polysomnography (PSG). All subjects were required not to take coffee, alcohol, and sedative hypnotic drugs prior to sleep study. PSG evaluation included airflow monitoring with thermocouple and/or nasal pressure, respiratory effort using piezo belts at the chest and abdominal positions, oxygen saturation using pulse oximetry, surface electrodes attached using standard techniques to obtain an electrooculogram, electromyogram of the chin. Sleep stages were defined according to Rechtshaffen and Kales’ criteria by a professional polysomnographic technologist. Hypopnea was defined as a reduction in the amplitude of airflow of at least 30% for ≥10 seconds, followed by either a decrease in oxygen saturation of 4% or at least 50% for ≥10 seconds, followed by either a decrease in oxygen saturation of 3% or signs of physiologic arousal (at least 3 seconds of alpha activity). Apnea hypopnea index (AHI), lowest saturation of oxygen (LSaO2), oxygen desaturation index 3 (ODI3), and ODI4 during sleep were calculated in each patient. AHI≥15 events/h was defined as OSA and AHI < 15 events/h as non-OSA for this study, as reported in recent studies from our center .^[18]^

### 2.3 Definition of obesity

Study populations were divided into nonobese and obese groups on the basis of body mass index (BMI). Obesity is defined as BMI ≥ 28 kg/m^2^.

### 2.4 Laboratory assessment

Samples of peripheral venous blood were collected in the morning after PSG at the sleep monitoring room of hypertension center by the same trained specialty nurse. Biochemical evaluation was measured by hospital laboratories using standard techniques within 2–3 hours after collection. Serum levels of d-LA, IL-1β, IL-6, and TNF-α were measured by commercial laboratories using sandwich-type enzyme immunoassay kit (Colorful Gene Biological Technology Co., Ltd, Wuhan, China) by personnel, who were blinded to the clinical characteristics of subjects. The reference value is 40 to 1600 μg/L for serum d-LA, 1 to 40 ng/mL for LBP, 2 to 80 ng/L for IL-1β, 1 to 20 ng/L for IL-6 and is 8 to 400 pg/mL for TNF-α, respectively. Intra-assay coefficients of variation range < 9% and interassay coefficients of variation <15%.

### 2.5 Data analysis

Before statistical analysis, normal distribution and homogeneity of the variances were evaluated. Data are expressed as means ± SD if normally distributed and median (interquartile) if not normally distributed. Subjects were divided into 4 groups, based on the presence or absence of obesity and OSA as nonobese non-OSA, nonobese OSA, obese non-OSA, and obese OSA group. Significant differences within groups were analyzed using ANOVA followed by post-hoc tests with LSD corrections for multiple comparisons and using Mann–Whitney *U* test. Pearson's correlation coefficients were used to assess the relationship between AHI, LSaO2, ODI3, and ODI4 and d-LA. Stepwise multiple linear regression analysis was used to test associations between d-LA and AHI, LSaO2, ODI3, and ODI4, after adjusting age, BMI and FBG. Value of *P* < .05 was considered to be statistically significant. The statistic analyses were performed using the statistics package for social science (SPSS version 19.0).

## 3 Results

As exhibited in Table 1, no significant differences emerged for baseline such as age, systolic and diastolic blood pressure, fasting blood glucose, hepatic and renal functions, and lipid profiles.

**Table 1:**
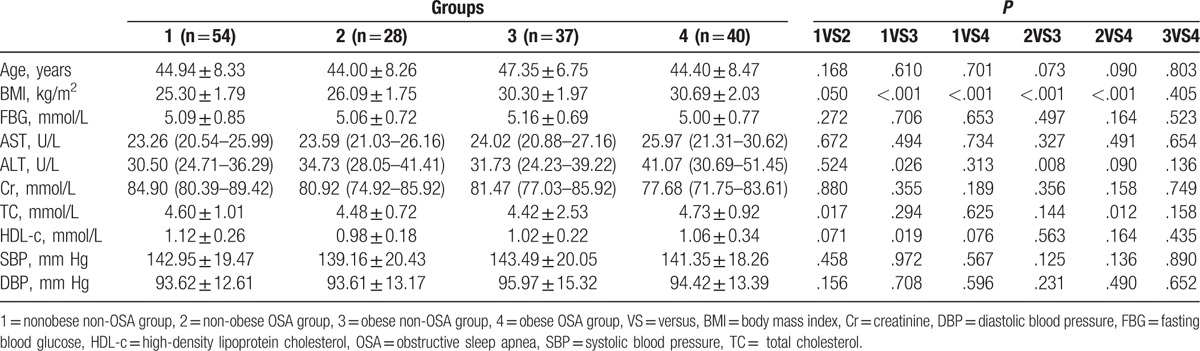
Comparison of characteristics on total subjects with and without OSA and obesity.

As expected as given in Table 2, either nonobese or obese OSA subjects displayed significantly higher AHI, ODI3, and ODI4, and significantly lower LSaO2, compared to non-OSA subjects. Interestingly, nonobese OSA group showed significantly higher serum d-LA than did nonobese non-OSA group [1374.35 (816–1735) vs 1166.43 (730–1815) μg/L, *P = *.018], than did obese non-OSA group [1374.35 (816–1735) vs 1188.75 (736–1557) μg/L, *P = *.045], and than did obese OSA group [1374.35 (816–1735) vs 1132.09 (680–1813) μg/L, *P = *.028], whereas serum LBP levels showed no differences within groups. Serum IL-1β was slightly higher in nonobese OSA group, but with statistical significance, than in nonobese non-OSA group (19.39 ± 4.67 vs 17.25 ± 3.66 ng/L, *P = *.041), than in obese non-OSA group (19.39 ± 4.67 vs 17.42 ± 3.79 ng/L, *P = *.047), and than in obese OSA group (19.39 ± 4.67 vs 17.53 ± 3.55 ng/L, *P = *.049), whereas other biomarkers, IL-6 and TNF-a did not show significant differences among groups.

**Table 2:**
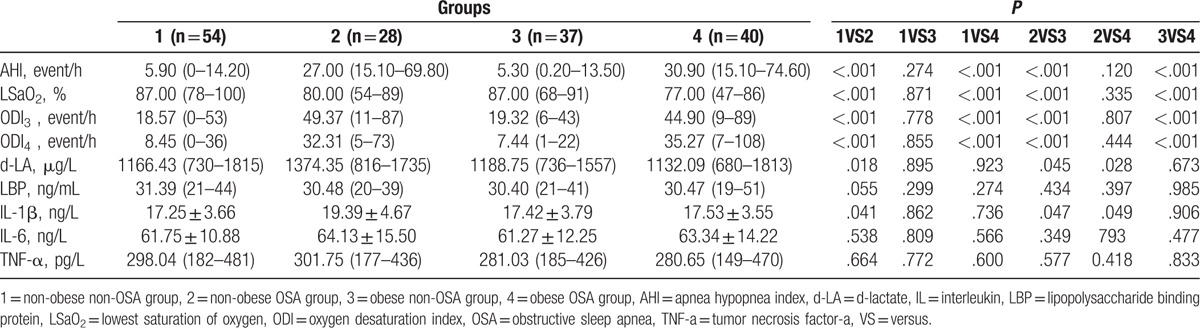
OSA, Intestinal and inflammatory parameters of subjects with and without OSA and obesity.

**Figure 1:**
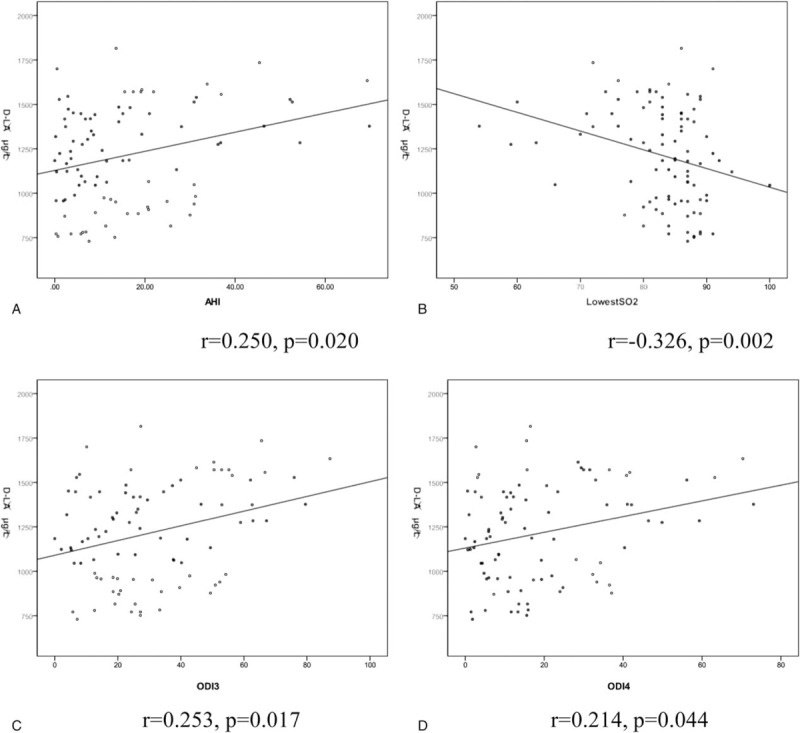
The correlationship between d-lactate and OSA parameters in nonobese subjects. OSA = obstructive sleep apnea.

On the basis of above observations, potential correlations were assessed between serum d-LA and AHI, LSaO2, ODI3 , and ODI4 in the nonobese subjects, and significant positive correlation between serum d-LA and AHI (*r* = 0.250, *P = *.020), ODI3 (*r* = 0.253, *P = *.017) and ODI4 (*r* = 0.214, *P = *.044) and significant negative correlation with LSaO2 (*r* = −0.326, *P = *.002) were observed, as exhibited in Fig. 1.

As shown in Table 3, to further explore whether AHI, LSaO2, ODI3, and ODI4 were independent predictors of serum d-LA levels, stepwise multiple linear regression analysis was performed with age, BMI and FBG included as potential confounders and observedly serum levels of d-LA were independently positively associated with AHI (*B* = 5.577, *P = *.022), and ODI3 (*B* = 4.550, *P = *.024) and negatively with LSaO2 (*B* = −12.234, *P = *.019).

**Table 3:**
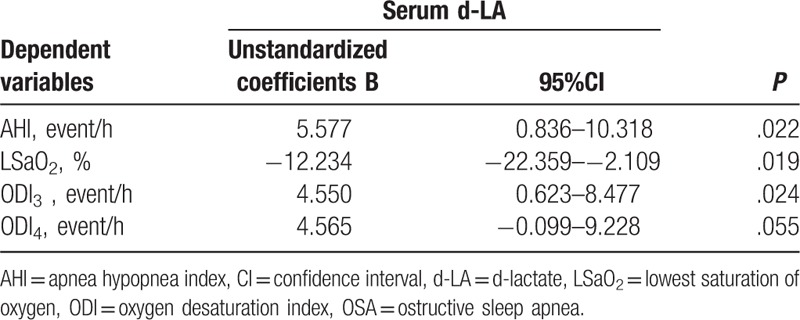
Stepwise linear regression between d-LA and OSA parameters in non-obese subjects.

As in Table 4, subjects were subdivided via the median of d-LA (1180.50ug/L) and the presence of obesity into four groups as nonobese lower d-LA, obese lower d-LA, nonobese higher d-LA and obese higher d-LA groups. Non-obese subjects with higher d-LA levels exhibited significantly higher serum LBP, IL-1β, IL-6, and TNF-a levels than did nonobese lower d-LA group [31.73 (22–44) vs 26.24 (20–39) ng/mL, *P = *.0018 for LBP; 19.80 ± 4.085 vs 16.24 ± 12.68 ng/L, *P* < .001 for IL-1β; 65.71 ± 13.62 vs 59.54 ± 11.75 ng/L, *P = *.046 for IL-6; 315.84 (186–481) vs 266.73 (177–376) pg/L, *P* < .001] and than did obese lower d-LA group [31.73 (22–44) vs 29.20 (19–37) ng/mL, *P = *.021 for LBP; 19.80 ± 4.085 vs 16.25 ± 2.89 ng/L, *P = *.004 for IL-1β; 65.71 ± 13.62 vs 58.95 ± 12.35 ng/L, *P = *.013 for IL-6; 315.84 (186–481) vs 268.87 (149–361) pg/L, *P = *.001]. Meanwhile, obese subjects with higher d-LA also sowed significantly higher serum LBP, IL-1β, and TNF-a levels than did nonobese subjects with lower d-LA [32.27 (25–51) vs 26.24 (20–39) ng/mL, *P = *.013 for LBP; 18.99 ± 3.87 vs 16.24 ± 12.68 ng/L, *P = *.009 for IL-1β; 310.41 (199–470) vs 266.73 (177–376) pg/L, *P = *.001 for TNF-a] and significantly higher serum LBP, IL-1β, IL-6, and TNF-a levels than did obese subjects with lower d-LA [32.27 (25–51) vs 29.20 (19–37) mg/mL, *P = *.021 for LBP; 18.99 ± 3.87 vs 16.25 ± 2.89 ng/L, *P = *.004 for IL-1β; 66.81 ± 13.90 vs 58.95 ± 12.35ng/L, *P = *.027 for IL-6; 310.41 (199–470) vs 268.87 (149–361) pg/L, *P = *.001], which might show that nonobese and obese subjects with higher serum d-LA levels are with more systemic inflammation, possibly suggesting involvement of gut in the inflammation in OSA. Nonobese subjects with higher d-LA [83 (54–92) vs 87 (66–100)%, *P = *.002] and obese subjects with higher d-LA [84 (61–89) vs 87 (66–100)%, *P = *.011] and obese subjects with lower d-LA levels 80 (47–91) vs 87 (66–100)%, *P* < 0.001] showed significantly lower SaO_2_ than did nonobese subjects with lower d-LA, possibly indicating that obesity per se and higher d-LA concentrations might suggest more hypoxic status in this specific population.

**Table 4:**
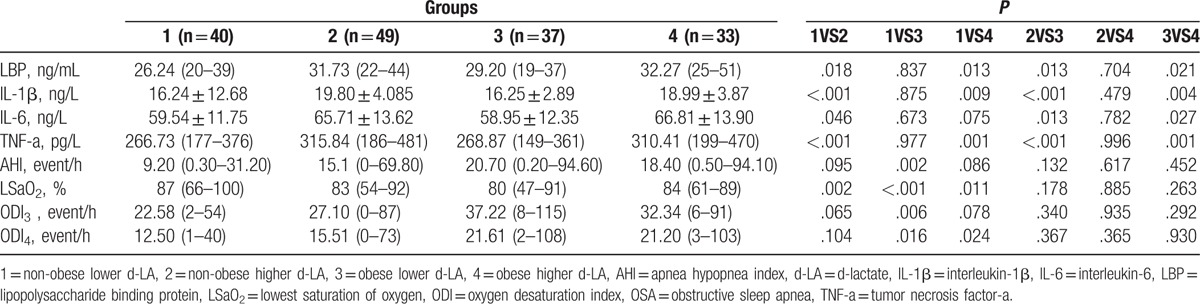
Comparison of OSA and inflammatory parameters in subjects stratified via the median of d-LA and the presence of obesity.

While subjects were subdivided via the median of LBP, similar results were also obtained. Data were not given.

## 4 Discussion

The intestine is not only an important organ of digestion and nutrient absorption, but also the one with immunomodulatory, endocrine, and mucosal barrier functions. Significant evidence exists that hypoxia contributes to disruption of intestinal barrier. However, still there are rare studies about effects of OSA on intestinal barrier, particularly in adults.^[5]^

Our primary findings encompass: (1) serum d-LA was significantly higher in middle-aged nonobese OSA males than in the nonobese and obese non-OSA ones (Table 2), and positively correlated with AHI, ODI_3_, and ODI_4_ and negatively with LSaO_2_, even after adjusting for age, BMI, and FBG (Fig. 1 and Table 3), 2). Nonobese and obese males with higher serum d-LA showed significantly higher LBP, IL-1β, IL-6, and TNF-α than did their counterparts (Table 4**)**.

### 4.1 Relationship between d-LA and OSA in the nonobese group

Low circulating levels of d-LA are found in healthy individuals, but in case of intestinal barrier function loss, these levels will rise as a consequence of increased translocation across the intestinal mucosa. Various studies proposed a relationship between circulating d-LA and intestinal permeability, for example, in patients undergoing open aortic surgery and ischemic colonic injury. Physiological sources of d-LA include dietary intake, gastrointestinal bacterial formation, and endogenous formation from methylglyoxal through glyoxalase system.^[15]^ Disturbances in these metabolic pathways, increased absorption by disrupted intestinal barrier and decreased secretion are possibly associated with increased d-LA in circulation. In gut ischemia reperfusion, resembling hypoxia reoxygenation in OSA, circulating d-LA has been indeed used as a well-established marker for disruption of intestinal barrier and intestinal hyperpermeability.^[16,17]^ Therefore, our results possibly suggest the existence of subclinically disrupted intestinal barrier and its hyperpermeability in condition of OSA. In this study, great care was taken to match groups in age, BMI, gender and FBG in order to establish a role of OSA, and thus while explaining increased d-LA, endogenous formation may not be considered. Nonetheless, possible involvement of small bacterial overgrowth (SIBO) may not be excluded, since in animal models exposed to hypoxia, density of main d-LA producing bacteria was increased.^[19,20]^ Indeed, it is evidenced that derangement of homeostasis between bacteria and the host in SIBO disrupts tight junctions and induces intestinal hyperpermeability.^[21]^ Furthermore, the existence of intestinal hyperpermeability in NAFLD patients was reported; importantly, over 80% of NAFLD patients with intestinal hyperpermeability showed SIBO.^[13]^ Animal models of NAFLD presented increased serum d-LA, compared to controls.^[22]^ Therefore, increased serum d-LA in the current study is possibly attributable to intestinal hyperpermeability due to subclinically disrupted barrier in OSA nonobese subjects, indicating that OSA may be a risk factor for subclinically increased intestinal barrier.

In obese population, the existence of increased intestinal permeability has been well evidenced, whereas the quantity of lactobacilli, predominant source of d-LA, was decreased.^[11,23]^ Thus d-LA may not severe as a biomarker for disruption of intestinal barrier in this specific population.

### 4.2 Relationship between LBP and OSA in nonobese subjects

Experimental data indicated that lipopolysaccharide (LPS), derived from gram-negative bacteria in the gut, plays a key role in driving systemic inflammation, insulin resistance, and fat mass development.^[24]^ However, application LPS detection is limited in routine clinical setting. Therefore, serum LBP serves as a LPS surrogate marker, whereas showed no significant differences between OSA and non-OSA groups, seemingly inconsistent with the findings from a previous study,^[5]^ and might be attributable to small sample sizes and different pathogenesis in pediatric and adult OSA. Based on limited information on roles of LBP in OSA, we are at present unable to explain the current findings. However, higher d-LA group had significantly higher LBP levels than did lower d-LA group.

### 4.3 D-LA and systemic inflammation

Another main observation, increased serum IL-1β, whereas not IL-6 and TNF-α, in nonobese OSA subjects compared to obese and nonobese non-OSA ones, extends the concept that OSA is a chronic low-grade systemic inflammation.^[25]^ Results from previous studies on potential links between these cytokines, OSAS, and CPAP therapy, have been conflicting, possibly generated from the fact that cytokine levels are influenced by a number of factors, such as inflammatory diseases including hypertension per se, life styles such as smoking, and some medications and the difference in subjects.^[26]^ In this study, grate care was given to match groups in terms of basic characteristics to establish the role of OSA. Thus, the limited sample size, small BMI ranges, normal blood glucose and age and potential effects of hypertension may explain the similarity on IL-6 and TNF-α in levels. We did, however, observe significantly higher levels of LBP, IL-1β, IL-6 and TNF-α in subjects with higher serum d-LA than in those with lower serum d-LA, indicating possible involvement of subclinical disruption of intestinal barrier and its hyperpermeability.

However, our study harbors several limitations. First, nature of the study, a cross-sectional observational one, does not allow us to draw a causal relationship between OSA and subclinical disruption of intestinal barrier. Second, as we did not directly assess serum LPS or biomarkers indicating disruption of intestinal barrier such as Zonula occludens, and thus some of results are suppositional. Third, study subjects are confined to middle-aged hypertensive males, and thus it may take further steps to generalize our results to OSA population of both genders. Finally, our results are from a single center, making chance and selection bias plausible explanations for our results and further validation is required to apply the results of this study to other populations.

In summary, OSA may affect intestinal barrier, facilitating subclinical disruption of intestinal barrier and translocation of bacterial products. Circulating d-LA levels are increased in some OSA individuals, possibly suggesting existence of subclinical disruption of intestinal barrier in in some middle-aged OSA males, which needs to be confirmed further.

## Acknowledgments

The authors thank all the participants who offered clinical data and blood samples to this current study and all the staffs of hypertension center.
